# Landscape dynamic network biomarker analysis reveals the tipping point of transcriptome reprogramming to prevent skin photodamage

**DOI:** 10.1093/jmcb/mjab060

**Published:** 2021-10-05

**Authors:** Chengming Zhang, Hong Zhang, Jing Ge, Tingyan Mi, Xiao Cui, Fengjuan Tu, Xuelan Gu, Tao Zeng, Luonan Chen

**Affiliations:** State Key Laboratory of Cell Biology, Shanghai Institute of Biochemistry and Cell Biology, Center for Excellence in Molecular Cell Science, Chinese Academy of Sciences, Shanghai 200031, China; University of the Chinese Academy of Sciences, Chinese Academy of Sciences, Beijing 100049, China; Unilever Research & Development Centre Shanghai, Shanghai 200335, China; State Key Laboratory of Cell Biology, Shanghai Institute of Biochemistry and Cell Biology, Center for Excellence in Molecular Cell Science, Chinese Academy of Sciences, Shanghai 200031, China; Unilever Research & Development Centre Shanghai, Shanghai 200335, China; Unilever Research & Development Centre Shanghai, Shanghai 200335, China; Unilever Research & Development Centre Shanghai, Shanghai 200335, China; Unilever Research & Development Centre Shanghai, Shanghai 200335, China; Bio-Med Big Data Center, Shanghai Institute of Nutrition and Health, University of Chinese Academy of Sciences, Chinese Academy of Sciences, Shanghai 200031, China; State Key Laboratory of Cell Biology, Shanghai Institute of Biochemistry and Cell Biology, Center for Excellence in Molecular Cell Science, Chinese Academy of Sciences, Shanghai 200031, China; School of Life Science and Technology, ShanghaiTech University, Shanghai 201210, China; Key Laboratory of Systems Health Science of Zhejiang Province, Hangzhou Institute for Advanced Study, University of Chinese Academy of Sciences, Chinese Academy of Sciences, Hangzhou 310024, China; Guangdong Institute of Intelligence Science and Technology, Zhuhai 519031, China

**Keywords:** single-sample network, tipping point, UVB irradiation, living skin equivalent model, time series data, skin lightening

## Abstract

Skin, as the outmost layer of human body, is frequently exposed to environmental stressors including pollutants and ultraviolet (UV), which could lead to skin disorders. Generally, skin response process to ultraviolet B (UVB) irradiation is a nonlinear dynamic process, with unknown underlying molecular mechanism of critical transition. Here, the landscape dynamic network biomarker (l-DNB) analysis of time series transcriptome data on 3D skin model was conducted to reveal the complicated process of skin response to UV irradiation at both molecular and network levels. The advanced l-DNB analysis approach showed that: (i) there was a tipping point before critical transition state during pigmentation process, validated by 3D skin model; (ii) 13 core DNB genes were identified to detect the tipping point as a network biomarker, supported by computational assessment; (iii) core DNB genes such as COL7A1 and CTNNB1 can effectively predict skin lightening, validated by independent human skin data. Overall, this study provides new insights for skin response to repetitive UVB irradiation, including dynamic pathway pattern, biphasic response, and DNBs for skin lightening change, and enables us to further understand the skin resilience process after external stress.

## Introduction

Skin is vulnerable to long-term exposure of environmental stressors like ultraviolet (UV) irradiation (Decean et al., 2016; Cavinato et al., 2017). Exposure to ultraviolet B (UVB) irradiation was reported to be the main reason for mutagenic and carcinogenic induction by sunshine (Afaq et al., 2005). UV light is classified as a ‘complete carcinogen’ because it is both a mutagen and a nonspecific damage agent, with dual characteristics of a tumor initiator and a tumor promoter (D'Orazio et al., 2013). Additionally, UVB irradiation can cause inflammation, immune suppression, apoptosis, DNA damage, and mitochondrial dysfunction (Yin et al., 2013; Decean et al., 2016; Zhang et al., 2017). Therefore, it is important to investigate the underlying mechanism of skin response to repetitive long-time UVB exposure to protect skin from photodamage.

Numerous studies investigated skin responses to UV irradiation from different viewpoints. Choi et al. (2010) conducted a transcriptome study to demonstrate the difference between UVB and ultraviolet A (UVA) in pigmentation. Huang et al. (2011) studied the gene expression adaptation at a series of time points after repetitive UV irradiation within one day. Malcov-Brog et al. (2018) studied the trade-off skin protection under different UV exposure frequencies to show the timer-controlled linkage between stress and pigmentation. Kim et al. (2013) adopted time series gene expression to investigate the senescence process and identified the genes linked to stage-specific pattern during senescence. Furthermore, there are a lot of time series data to understand skin barrier formation process (Taylor et al., 2009), elicitation of allergic contact dermatitis (Pedersen et al., 2007), skin burn injury (Xu et al., 2018), etc.

However, previous studies mainly focused on one or more time points at individual gene expression level, ignoring the whole dynamic process. Many studies have suggested that the progression of complex disorders was not always smooth, but occasionally abrupt, indicating the existence of tipping point (Chen et al., 2012; Yang et al., 2018). Generally, the skin maintains renewal and keeps homeostasis at system level and the homeostasis is stable in a normal state. Under external stressor stimulation, the homeostasis might be disrupted and reach a predisorder state (i.e. tipping point), without significant change in phenotype compared with normal state. The predisorder state is reversible and can turn back to the normal state easily. However, if the biological system crosses the tipping point, the skin will deteriorate rapidly, eventually leading to a disorder state. At the tipping point, a dominant gene group named dynamic network biomarkers (DNBs) play a driving role during the state transition (Chen et al., 2012; Liu et al., 2021a). Obviously, determining the tipping point is important for prevention strategy. Both theoretically and experimentally, DNB genes have been shown to provide early-warning signals for diseases (Chen et al., 2012; Li et al., 2014; Zeng et al., 2014; Yang et al., 2016, 2018; Liu et al., 2021b).

**Figure 1:**
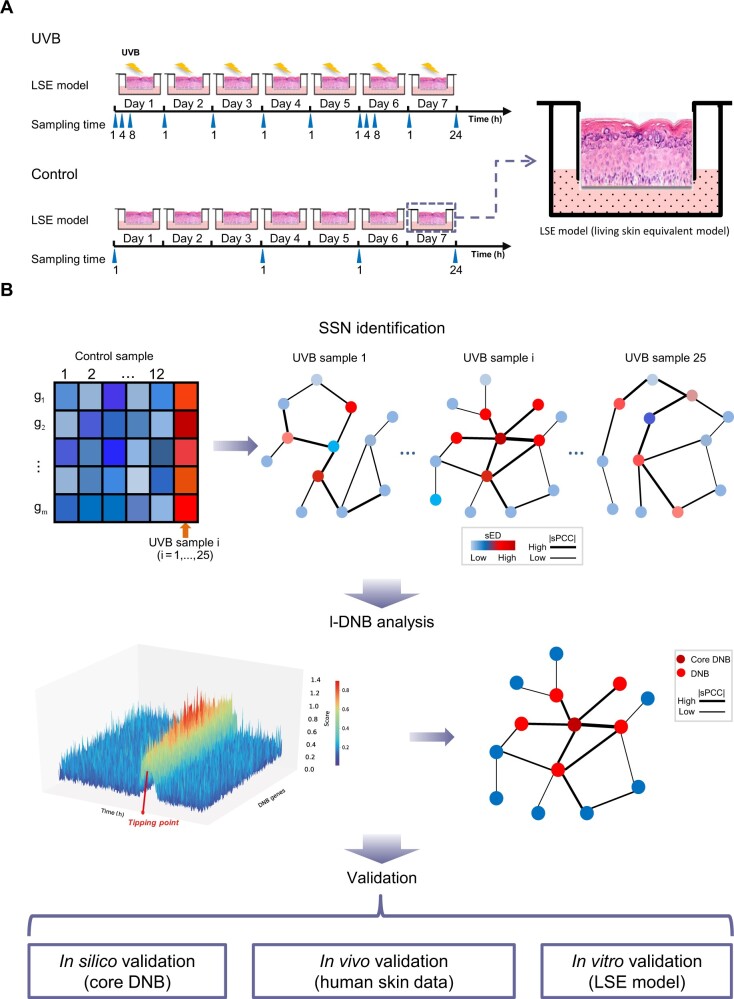
Framework of LSE experiment and l-DNB data analysis. (**A**) Schematic diagram of the experimental design on 3D skin model. (**B**) The flowchart of landscape l-DNB method to identify DNB genes from a single sample and validation from different aspects.

Here, to reveal the tipping point of skin response to UVB irradiation, we conducted landscape DNB (l-DNB) analysis of time series transcriptome data on the advanced 3D skin model, pigmented living skin equivalent (LSE) model, under repetitive UVB irradiation (Figure  1A and B). We found that (i) a biphasic response to UVB irradiation, acute response, and adaptation response with adaptation stage as the tipping point, was interfered after intervention; (ii) core DNB genes including CALML5, TGFB1, BRCA1, HNRNPD, PCNA, COL7A1, CTNNB1, ERCC2, COL4A2, PSMB10, SFN, ITGB1, and NF-κB1 were prioritized as early-warning signals of skin photodamage; (iii) COL7A1 and CTNNB1 could effectively predict the prognosis for UVB irradiation, supported by independent *in vivo* skin data. Collectively, our study combining wet LSE model with dry l-DNB model investigated the tipping point of transcription reprogramming during UVB irradiation and identified core DNB genes, which not only accurately predicted the skin lightness but also effectively prognosed in independent clinical trial. The results provide new insights into skin response to UVB irradiation and novel network biomarkers for skin protection.

## Results

### UVB induces continuous decline of skin lightness and dynamic changes at gene and pathway levels

**Figure 2:**
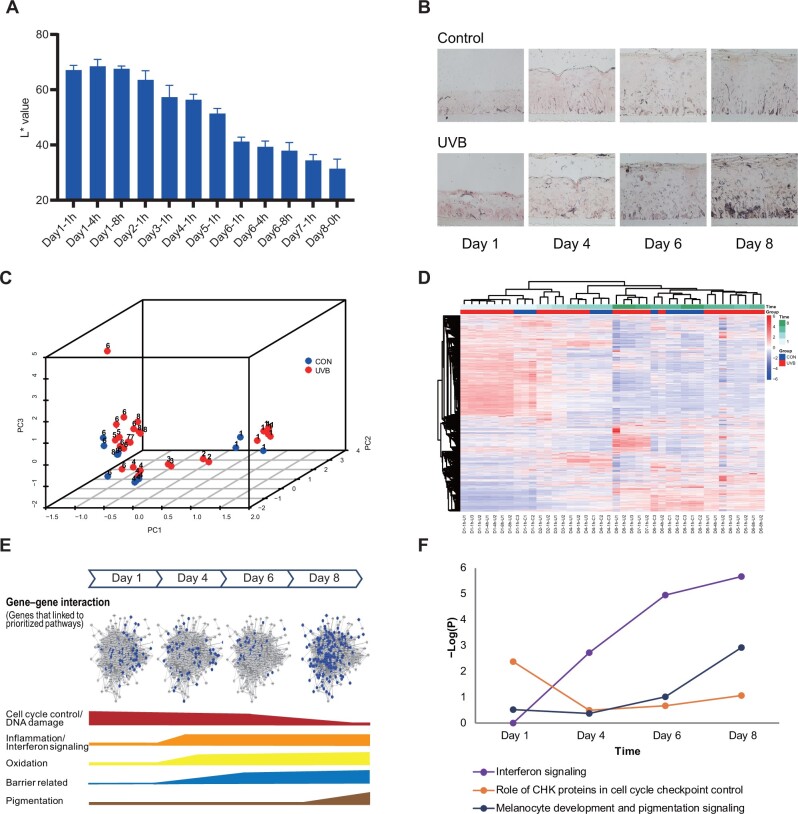
Skin transcriptome changes during UVB irradiation revealed by differential expression analysis. (**A **and** B**) Repetitive UVB irradiation results in a consecutive decline of L* (**A**) and increased melanin content (**B**). (**C**) PCA of whole-genome gene expression. Blue nodes: samples from control group; red nodes: samples from UVB group. Number on each node: sampling time (day). CON, Control. (**D**) Heatmap of DEGs for all samples. D1-1h-U represents the sample from UVB group at first hour of Day 1. D1-1h-C represents the sample from control group at first hour of Day 1. (**E**) Dynamic pattern of global pathways involving DEGs and the specific pathway involvement. Blue node on network: DEGs at corresponding time point. The height of bar: involvement based on *P*-value from IPA pathway analysis at corresponding time. (**F**) Dynamic involvement of prioritized pathways over time.

To study the dynamic response of skin to UVB exposure, two parallel experiments on LSE model, with/without UVB irradiation, were conducted simultaneously (Figure  1A). Transcriptome data and phenotypes (skin lightness: L*) at multiple time points were obtained (see Materials and methods section). The repetitive UVB irradiation resulted in a continuous decline of L* (Figure  2A; Supplementary Figure S1) and increase of melanin content (Figure  2B), indicating a skin lightness change after UVB irradiation. Principal component analysis (PCA) of gene expression presented that both UVB and control samples showed a temporal tendency (Figure  2C). There was a slight difference between UVB and control groups before Day 6. There was an inflection point around Day 6, which indicated the potential existence of a critical transition of skin response process of UVB irradiation. Moreover, hierarchical clustering analysis showed that samples were classified into two broad clusters by time roughly (Supplementary Figure S2). Samples from Day 1 to Day 3 were in the same cluster and samples from Day 4 to Day 8 were in another, indicating the early phase and later phase differing in gene expression after UVB irradiation.

Conventional pairwise comparison was conducted to investigate differentially expressed genes (DEGs) induced by UVB at each sampling time point. Overall, there were 1431 DEGs on Day 1, 1386 DEGs on Day 4, 4879 DEGs on Day 6, and 1996 DEGs on Day 8. Obviously, the number of DEGs was not consistently increased with time due to accumulative UVB irradiation but fluctuated. The expression heatmap of DEGs also presented a time tendency of skin samples under UVB exposure (Figure  2D). Furthermore, biological pathways involved by DEGs at each time point also showed a dynamic pattern (Figure  2E). On Day 1, DEGs were involved in DNA damage and cell cycle-related pathways like ATM signaling and cell cycle control pathways. On Day 4, DEGs were involved in inflammation-related pathways such as STAT3 pathway and interferon signaling pathway. On Day 6, oxidative pathways such as ROS production pathway and AHR pathway were involved as well. On Day 8, melanocyte development and pigmentation signaling pathway showed up. Although some pathways, like interferon signaling pathway, were involved in both early and middle time, the involvement/enrichment significance within DEGs was different at different time points (Figure  2F). These prioritized pathways with their related genes/networks demonstrated the dynamic functional changes during UVB irradiation, indicating the potential transition from cell proliferation to pigmentation.

### The tipping point of skin response to UVB irradiation identified by l-DNB

**Figure 3:**
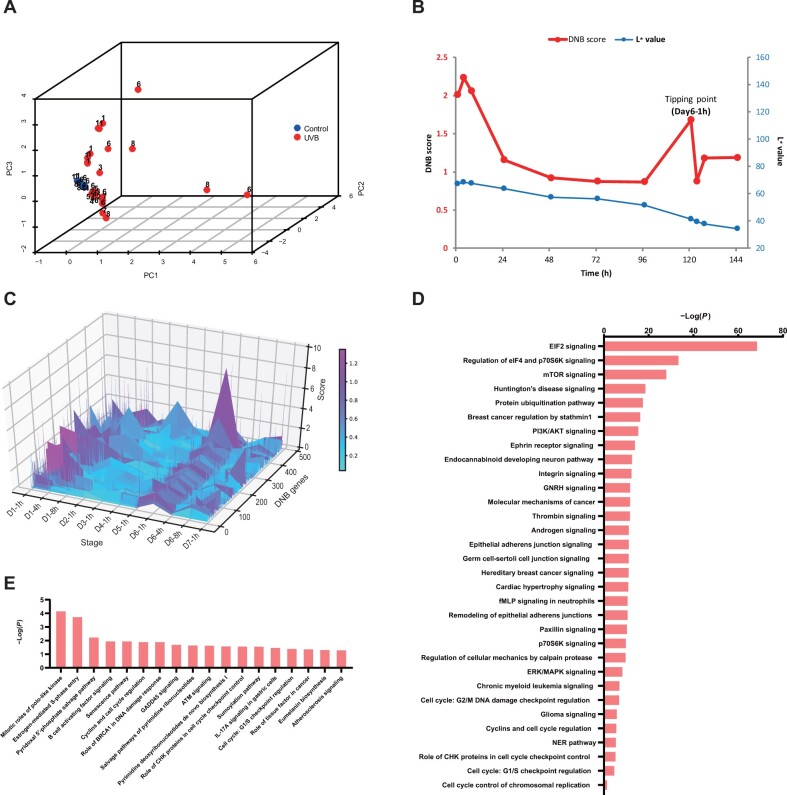
Tipping point of skin transcriptome dynamics during UVB irradiation identified by l-DNB analysis. (**A**) PCA based on SSN edge statistics. Blue nodes: samples from control group; red nodes: samples from UVB group. Number on each node: sampling time (day). (**B**) Average DNB scores for UVB samples. (**C**) The landscape of DNB genes for UVB samples. (**D**) DNB genes-involved pathways. (**E**) TP-DEGs-involved pathways. −Log(*P*) is the pathway enrichment/involvement significance through IPA analysis.

To decipher the underlying dynamic process after UVB irradiation, the l-DNB approach was adopted and improved to incorporate network information for interpreting systemic dynamics. First, the single-sample network (SSN) approach (Liu et al., 2016) was used to identify differential networks induced by UVB at each time point. PCA analysis on the network statistics of SSNs showed that the UVB group was distinguished from the control group; by contrast, only incorporating gene expression information could not detect such essential discrimination (Figure  3A). Interestingly, the distribution of samples from the first hour of Day 6 (Day6-1h) fluctuated greatly, consistent with the above observation of potential tipping point. DNB scores as index for tipping point identification presented two peaks: one on Day 1 and the other at the first hour of Day 6 (Figure  3B). The change on Day 1 mainly demonstrated the acute response to UVB exposure from the pathways involved by DEGs on Day 1. The change on Day 6 indicated the adaptation phase before phenotype change, since the skin phenotype showed obvious difference at the end of the experiment and pigmentation-related pathways showed up as well. Therefore, the second peak (Day6-1h) was defined as the tipping point of skin lightness for skin response to repetitive UVB irradiation. The tipping point and DNB genes have been robustly identified (Supplementary Figure S3), and DNB genes (480 genes) at the tipping point were prioritized by ranking each gene’s DNB score and existence in fused SSN at the tipping point (Materials and methods Section). The landscape of DNB genes at each time point was shown in Figure  3C and DNB genes with higher scores at the tipping point can be early-warning signals to indicate the imminent skin condition transition.

DNB genes were significantly involved in plenty of pathways like EIF2 signaling, mTOR pathway, PI3K pathway, and integrin pathways (Figure  3D). Upstream factors including transcription factors were predicted to decipher the trigger effect of DNB genes for gene expression change. Using *P*-value as index, the top five transcription regulators were BRCA1, TP53, E2F4, MYC, and TWIST1. BRCA1, TP53 (van der Pols et al., 2006), E2F4 (Jones et al., 1997), and MYC (Bretones et al., 2015) were reported to act on cell proliferation and influence skin homeostasis and TWIST1 (Hasei et al., 2017; Weiss et al., 2012) was reported to regulate MMP and TIMP expression, indicating the effect of DNB genes for skin homeostasis.

To capture gene regulation at time level, DEGs between tipping point and its neighbor time point (TP-DEGs) were identified for enrichment analysis. TP-DEGs over time were involved in pathways including senescence pathway that is a cell response to stress, GADD45 signaling and ATM signaling that are DNA damage and repair-related pathways, Eumelanin biosynthesis that can potentially influence lightening, and some other cell cycle-related pathways (Figure  3E; Supplementary Figure S4).

### Identification of DNB network and core DNB genes responding to UVB irradiation

**Figure 4:**
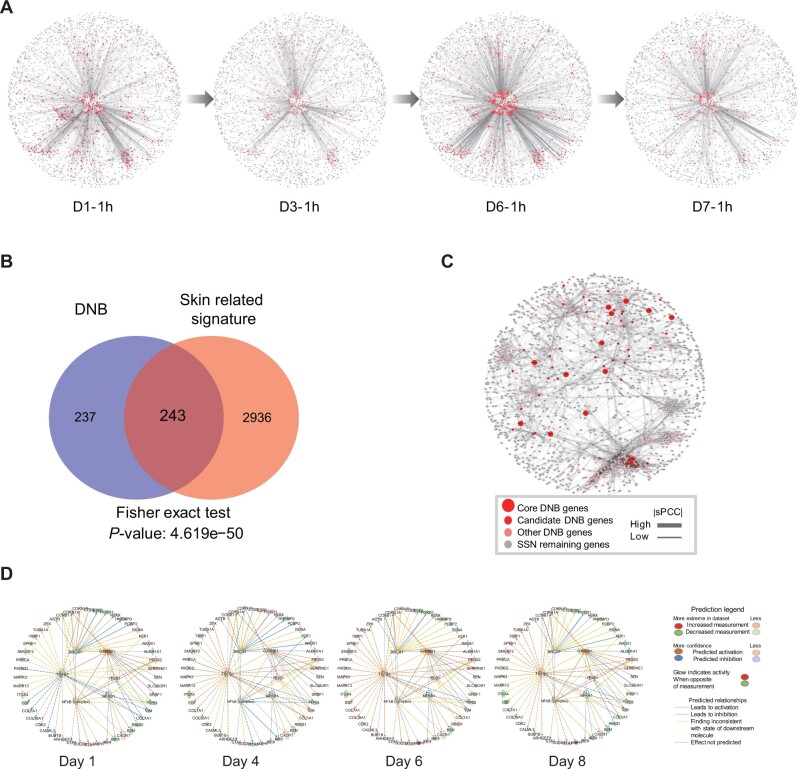
Identification of DNB network and core DNB genes responding to UVB irradiation. (**A**) SSN overview among all time points (red: DNB score for each gene). (**B**) Overlap between DNB and skin-related signature genes. (**C**) A dynamic endogenous core DNB network. (**D**) Upstream factor of DNB genes among core DNB genes with their regulated DNB genes. Green: downregulated at the specific time. Orange node: upregulated at the specific time.

Dynamic change of SSN over time demonstrated the network evolution induced by UVB irradiation. SSN dynamic change showed that there was a denser network at the tipping point (Figure  4A; Supplementary Figure S5), indicating accurate and robust signals. Differential network between tipping point and its adjacent time points was identified (Supplementary Figure S6). The identified network presented that numerous edges of network at the tipping point were absent at the time points before or after tipping point. The change of edges indicated that the network rewired when crossing the tipping point.

The first-order neighbors of skin signatures (Choi et al., 2010) on the protein‒protein interaction (PPI) and themselves were extracted as skin-related genes for prioritizing core DNB genes. The intersection between DNB genes and skin-related genes was extracted. DNB genes were significantly enriched in skin-related genes, suggesting that DNB genes were strongly associated with prior-known skin signatures (Figure  4B). Genes with the node degree greater than the average node degree are prioritized as differential network genes at tipping point (TP-DNGs) for further analysis.

Combining all information from above obtained SSNs, DNB genes, TP-DEGs, and TP-DNGs, with functional anchors from the collected prior-known skin signatures (Choi et al., 2010), 13 core DNB genes CALML5, TGFB1, BRCA1, HNRNPD, PCNA, COL7A1, CTNNB1, ERCC2, COL4A2, PSMB10, SFN, ITGB1, and NF-κB1 were prioritized. Supposing that these core DNB genes play a leading role in the dynamic process of UVB irradiation on skin, their first-order neighbor DNB genes should also be associated and connected to form the core DNB network that regulates skin deterioration under UVB exposure. As a clearly organized illustration, the core DNB genes and their first-order neighbor DNB genes in SSN were displayed in Figure  4C. The intersection of these DNB genes and TP-DEGs (or TP-DNGs) was taken as candidate DNB genes. And the core DNB genes and candidate DNB genes reflected the layer composition of DNB genes on the network (Liu et al., 2012; Jiang et al., 2020).

Among core DNB genes, BRCA1, CTNNB1, TGFB1, ITGB1, and NF-κB1 were upstream factors, which were predicted by Ingenuity Pathway Analysis (IPA) using DNB genes (Figure  4D). They regulated gene expression during UVB irradiation. The gene expression changes at four time points induced by UVB demonstrated that CTNNB1, ITGB1, and NF-κB1 were consistent at four time points, while TGFB1 was downregulated for short time but upregulated starting from Day 4 and BRCA1 was first downregulated and upregulated starting from Day 6. Interestingly, these genes’ expression levels were fluctuated over time within the UVB group. For example, CTNNB1 was downregulated at the tipping point and then upregulated at later time points. Moreover, these genes were reported to interplay with numerous genes linked to skin benefit, such as COL1A1, COL7A1, and TIMP1 acting on collagen synthesis and degradation (Valizadeh et al., 2015; de la Fouchardiere et al., 2019).

### Validation of the tipping point and core DNB genes for skin response to UVB irradiation

*In silico*, *in vitro*, and *in vivo* validations were conducted to verify the importance of tipping point for skin protection and consolidate the linkage between core DNB genes and skin lightness.

**Figure 5:**
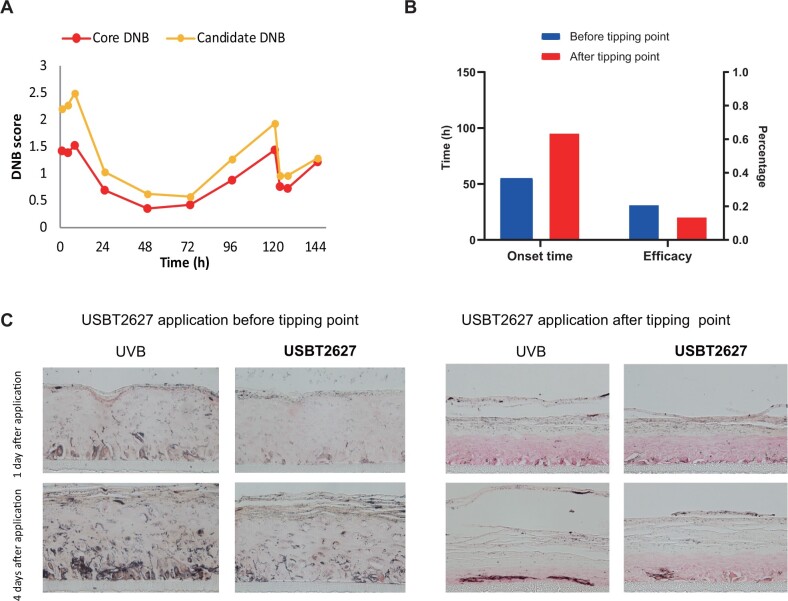
Assessments of the tipping point and core DNB genes of skin response to UVB irradiation. (**A**) DNB scores calculated by just core DNB genes or by all candidate DNB genes. (**B**) Comparison of the onset time and efficacy between USBT2627 treatments before and after the tipping point. Onset time: the time when skin sample showed significant increase of L*. Efficacy: normalized increasement of L* in USBT2627 group compared to UVB group at onset time. (**C**) Melanin staining between USBT2627 treatments before and after the tipping point.

For *in silico* validation, the DNB score curve calculated using core DNB genes showed consistent tendency with the one using all genes (Figure  5A). For *in vivo* validation, a skin lightening agent (USBT2627) was applied to reverse UVB-induced skin phenotype change. USBT2627 has been demonstrated to have a skin lightening effect through numerous internal *in vitro and in vivo* studies. Here, an independent human skin dataset (two groups: with/without USBT2627, with 19 samples in each group) with phenotype changes (L*) from clinical trial was adopted for *in vivo* validation. Using L* value as a phenotype factor of human skin with UVB irradiation, the modified risk/survival curve corresponding to core DNB genes was calculated, and several core DNB genes had consistently significant *P*-values as listed in Supplementary Tables S1 and S2. In particular, COL7A1, CTNNB1, and ERCC2 had a good prognosis performance as molecular biomarkers in both the placebo and active groups, which could be targeted for subsequent biomarker research for skin protection (Supplementary Figure S7).

An independent *in vitro* validation using USBT2627 was conducted (Supplementary Figure S8) to demonstrate the importance of skin protection before tipping point. USBT2627 significantly changed L* on Day 2 after application if added before the tipping point, while there were no significant changes if added after the tipping point. Also, if USBT2627 was added before the tipping point, L* change occurred in a shorter time with a better efficacy (Figure  5B). This fact strongly supported that the skin system has reached a critical state that is difficult to be reversed after the tipping point, and similar result from examing the melanin content (Figure  5C) proved the critical point for human skin response to UVB irradiation.

## Discussion

The progress of human skin under UVB irradiation is a dynamic process affected by various factors from environment. Due to the complexity of the skin regulation system, the conventional analysis on a single time point cannot identify high-risk individuals and predict the disease or abnormal skin state induced by UVB irradiation. Thus, there is a strong demand for new analysis on dynamic perspective.

Here, the LSE model was adopted to mimic skin epidermis. The DEGs and involved pathways showed a dynamic pattern over time, indicating that changes of both genes and pathways after UVB irradiation were nonlinear, not consecutively increased or decreased. The dynamic pattern successfully mimicked the real skin exposed to UVB irradiation, building the confidence for the application of LSE model for other related scientific research. Moreover, skin showed a biphasic response to repetitive UVB exposure, and a group of DNB genes that play a driving role in the lightening process of skin exposed to UVB were identified as indication for tipping point. As solid evidence for such tipping point and DNB genes, different validations have been carried out, verifying the existence of tipping point during UVB irradiation on human skin and the efficiency of core DNB genes on the personalized risk prediction of skin response during UVB irradiation.

The function and upstream analysis of DNB genes revealed that top upstream factors were linked to skin homeostasis that decipher the vital effect of tipping point for skin protection. Furthermore, a gene module consisting of 13 genes was prioritized as a network biomarker providing early-warning signals for skin lightness induced by UVB. These genes were not well-known pigmentation-related genes, while some genes were highly associated with proliferation, like PCNA, or cell cycle or apoptosis, like NF-κB1, indicating the high correlation between cell proliferation and later skin lightening change. Besides, core DNB genes were compared with public domain melasma study (Kang et al., 2011). Core DNB gene TGFB1 interacted with VEGFA, which was significantly changed in melasma through STRING PPI network. ITGB1 interacted with COMP and VEGFA as well. Furthermore, CTNNB1 was linked to genes like DCT and HMGCS1, which were related to melanin biosynthetic process and lipid metabolic process reported in melasma study.

Besides, pathways involving DEGs at different time points through conventional analysis were compared with pathways involving DNB genes through IPA. The results showed that the pathways involving DNB genes were more like those involving DEGs on Day 8. The results indicated that the tipping point and DNB genes identified by l-DNB analysis indeed captured early information for later biological process changes.

Collectively, this work adopted the l-DNB method to determine the critical point of human skin under UVB irradiation. The core DNB genes were found to play a driving role in the development of skin damage process, providing a new perspective for studying the molecular network mechanism of human skin protection against UVB exposure. Furthermore, the personal early-warning signals can be measured by core DNB genes and serve as potential network biomarkers for UVB risk prediction.

## Materials and methods

### LSE model and model treatment

The pigmented LSE model is a derived 3D skin model consisting of normal human keratinocyte and melanocyte (Mi et al., 2019), supplied by Biocell. The model is structurally similar to human epidermis including basal, spinous, and granular layers as well as stratum corneum (Figure  1A).

The LSE model was exposed to a repetitive UVB irradiation (50 mJ/cm^2^, one time per day, lasting 7 days) to mimic skin exposure to UVB in real life (Figure  1A). Model materials were collected at 1, 4, and 8 h after 1st (Day 1) and 6th (Day 6) irradiation, 1 h after 2nd, 3rd, 4th, 5th, 7th irradiation, and the end of the experiment (24 h after 7th irradiation, labeled as Day 8). Parallelly, the control group without any challenge was sampled on Day 1, Day 4, Day 6, and the end of the experiment (Day 8) (Figure  1A). For RNA-sequencing analysis, there were three replicates for 1 h of Day 1, Day 4, Day 6, and the end of experiment (Day 8) in order to carry out conventional DEG analysis and two replicates for each of other time points. Two replicates at each time point were collected for melanin staining. Three replicated samples at each time point were collected for L* measurement. L* is used to indicate perceptual lightness. The L* value was measured using a CM600d spectrophotometer fitted with a 3-mm target mask (Konika-Minolta). For each sample, the lightness was repeatedly measured three times, and the mean value was calculated for comparison. Meanwhile, collected samples were sent to Berry Genomics for RNA-sequencing.

### RNA-sequencing and DEG and pathway analysis

RNA-sequencing was performed using Illumina NovaSeq6000 platform (150PE). Prebase quality was assessed with FastQC software. HISAT2 was applied to align raw files to the human genome. RSEM was used to quantify reads to cell counts and fragments per kilobase of transcript per millions mapped reads (FPKM). Statistical analysis was applied to test the DEGs between groups. Ingenuity Pathway Analysis software (QIAGEN^®^ Bioinformatics) was applied to pathway analysis. The *P*-value of pathway analysis was used as indication for pathway involvement.

### l-DNB analysis

The analysis pipeline of the time series data generated by the LSE model was designed and illustrated as Figure  1B. Generally, the l-DNB method (Liu et al., 2019) was modified and improved to incorporate both molecule and network information of each sample to identify tipping point. Differential expression analysis and differential network analysis between time points before and after tipping point were conducted to determine core DNB genes and their involved network (Hu et al., 2020).

First, the control group samples were used as reference samples to construct SSN (Liu et al., 2016; Guo et al., 2020) for each given sample in the UVB group. Second, DNB score was calculated based on constructed SSN at each time point to identify tipping point under UVB irradiation according to three conditions of DNB theory (Chen et al., 2012; Zeng et al., 2013; Liu et al., 2014) and to build the final l-DNB model. Third, differential expression analysis and differential network analysis (Yu et al., 2015; Hu et al., 2018) between samples before and after tipping point were performed. Finally, the DNB regulatory network was reconstructed from SSNs of all samples by linking DNB genes and prior-known skin characteristic genes as anchors (Hu et al., 2020), and then core DNB genes were prioritize for further validation. In detail, the calculation of l-DNB method includes the following two steps.

*SSN construction*. Reference network was built to identify SSN after UVB irradiation at each time point (Liu et al., 2016). The edge of reference network was constructed by the correlation (Pearson correlation coefficient, PCC) between genes using their expression values

$${\mathrm{PCC}}_{n}\left(x,\;y\right)=\frac{\sum_{i}^{n}\left(x_{i}-\bar{x}\right)\left(y_{i}-\bar{y}\right)}{\sqrt{\sum_{i=1}^{n}{\left(x_{i}-\bar{x}\right)}^{2}}\sqrt{\sum_{i=1}^{n}{\left(y_{i}-\bar{y}\right)}^{2}}}\;\;\;\;\;$$

where $x_{i}$ and $y_{i}$ are the expression levels of genes x and y in the $i_{th}\;$reference sample, respectively, and $\bar{x}$ and $\bar{y}$ are the average expression levels for genes *x and y* in reference samples with a sample size of *n*. Here, control group samples were reference samples for UVB group. Then after new sample d was added to reference samples, a new PCC of the genes x and y was recalculated using Equation (1) based on the n+1 samples, which is expressed as ${\mathrm{PCC}}_{n+1}\left(x,\;y\right)$.

The influence of the new sample d is mainly reflected in the change of PCC. Therefore, the single-sample PCC (sPCC) between genes *x and y* for sample *d* against the *n* reference samples is defined as

$$\mathrm{sPC}C_{n}\left(x,\;y\right)={\mathrm{PCC}}_{n+1}\left(x,\;y\right)-\mathrm{PC}C_{n}\left(x,\;y\right)\;\;\;\;\;$$

The ${\mathrm{sPCC}}_{n}\left(x,\;y\right)$ follows the volcano distribution (Liu et al., 2016), which approximates the normal distribution when *n* is sufficiently large. Statistical hypothesis test (*Z*-test or *U*-test) was applied to test whether gene *x* and gene *y* were significantly correlated at the single-sample level.

*Construction of DNB landscape.* The tipping point identification with DNBs by l-DNB method is mainly based on three conditions of original DNB theory (Chen et al., 2012; Liu et al., 2014, 2019): (i) the expression deviation of each gene inside the module (${\mathrm{ED}}_{\mathrm{in}}$, expression deviation in the module) fluctuates strongly; (ii) the expression correlation among genes inside the module (${\mathrm{PCC}}_{\mathrm{in}}$, average absolute PCC among inner genes of a module) dramatically increases; and (iii) the expression correlation of the genes between inside and outside of this module (${\mathrm{PCC}}_{\mathrm{out}}$, average absolute PCC between inner and outer genes of a module) dramatically decreases.

Target gene and its first-order neighbors in SSN were regarded as a local module. According to three statistical conditions of DNB theory, the local DNB score for each gene in sample *d* can be defined as follows:

$$I_{s}\left(x\right)=\mathrm{sE}D_{\mathrm{in}}\frac{\mathrm{sPC}C_{\mathrm{in}}}{\mathrm{sPC}C_{\mathrm{out}}}\;\;\;\;\;$$

where $I_{s}\left(x\right)$ is the score of the local module of gene *x* in the single sample *d*. Here, the deviation of gene *x* expression in a single sample against its expression in the reference samples, namely single-sample expression deviation (sED), was defined as

$$\mathrm{sE}D_{\mathrm{in}}=\frac{1}{1+n_{x_{d}}}\left[\mathrm{sED}\left(x\right)+\sum_{y\epsilon N_{x_{d}}}s\mathrm{ED}\left(y\right)\right]\;\;\;\;\;$$

 

$$\mathrm{sED}\left(x\right)=|x_{d}-\bar{x}|\;\;\;\;\;$$

where $x_{d}$ is gene expression of *x* in new sample *d* and $\bar{x}$ is the average expression of gene *x* in the *n* reference samples. $N_{x_{d}}$ is the gene set consisting of first-order neighbors of gene *x* in the SSN constructed by sample *d* and reference samples. $n_{x_{d}}$ is the gene number of first-order neighbors of gene *x* in the SSN of sample *d*. The Equation (4) represents average differential deviation of all $\left(1+n_{x_{d}}\right)$ genes’ expression in the local module of gene *x* for sample *d* against the *n* reference samples.

The correlation between genes within the module and correlation between inside and outside of module in SSN of sample *d* were similarly calculated, named as ${\mathrm{sPCC}}_{\mathrm{in}}$ and ${\mathrm{sPCC}}_{\mathrm{out}}$, respectively.

$$\mathrm{sPC}C_{\mathrm{in}}=\frac{1}{n_{x_{d}}}\sum_{y\epsilon N_{x_{d}}}s\mathrm{PC}C_{n}\left(x,\;y\right)\;\;\;\;\;$$

where ${\mathrm{sPCC}}_{\mathrm{in}}$ is the average value of ${\mathrm{sPCC}}_{n}$ between gene *x* and its first-order neighbors

$$\mathrm{sPC}C_{\mathrm{out}}=\frac{1}{K_{x_{d}}}\sum_{\begin{array}{c} y\epsilon N_{x_{d}}\\ z\epsilon M_{x_{d}} \end{array}}s\mathrm{PC}C_{n}\left(y,\;z\right)\;\;\;\;\;$$

where ${\mathrm{sPCC}}_{\mathrm{out}}$ is the average of ${\mathrm{sPCC}}_{n}$ between the first-order neighbors and second-order neighbors of gene x in the SSN of sample *d* constructed by sample *d* and reference samples. $M_{x_{d}}$ is a gene set consisting of the second-order neighbors of gene x in the SSN of sample *d*. And $K_{x_{d}}$ is the number of network connections between $N_{x_{d}}$ and $M_{x_{d}}$ in the SSN. Only genes having at least three first-order neighbors and one second-order neighbor in the network topology of the SSN were selected for l-DNB analysis.

The DNB score of each sample was the average DNB score of top *K* genes in the SSN. If there were multiple samples at the same time point, the average DNB score of all samples at the time point was used as the DNB score of the time point. In the present study, according to the number of genes in each SSN and the landscape of each gene’s local DNB score, *K* = 600 was used. Actually, the DNB score showed a similar tendency when trying different *K* values. And the tipping point was identified by selecting the largest DNB score. DNB genes were identified by retrieved genes on the SSN at the identified tipping point. In detail, to ensure the robustness of the identified DNB genes, we took the gene whose local DNB score ranked in top *K* in at least two samples at the tipping point as the DNB gene of the tipping point.

### Core DNB gene and network identification by differential network analysis

Network edges/features presenting in at least two SSNs at each time point were retained, which not only ensured the robustness of selected network features but also considered the specific information in different samples. Using fused SSNs at every time point as the background network, the rewiring of DNB-related networks was reconstructed. For a fusion network at a given time point, the DNB score of the gene appeared in this network was represented by the mean local DNB score in all SSNs at this time point, and the scores of other genes were set to 0.

Next, the differential network analysis was conducted using those SSNs at the tipping point and its adjacent time points. Edges in differential networks were newly appeared edges or disappeared edges between SSNs before tipping point and at tipping point (i.e. the prior differential network) or between SSNs at tipping point and after tipping point (i.e. the posterior differential network). Depending on the tipping point-relevant differential networks, we can obtain hubs, i.e. genes/nodes with large node degree in the network, which are considered to play an important role in function due to their hub/center position in the rewiring network. The nodes with high node degree in differential networks were ranked and prioritized for further analysis, e.g. the genes with higher degrees than average value were selected as TP-DNGs.

Furthermore, DNB genes were overlaid with skin-related genes extracted from knowledge-based skin signature genes (Choi et al., 2010), and intersection genes between DNB genes and skin signature genes were core DNB genes, which were also TP-DEGs or TP-DNGs. In details, core DNB genes were prioritized with the following steps: (i) the skin signatures and first-order neighbors based on the PPIs were retrieved as skin-related genes; (ii) the intersection genes between DNB genes and skin-related genes were selected, and the overlap significance was tested using Fisher Exact test; and (iii) the intersection genes between DNB genes and skin signature genes were selected as core DNB genes if they were TP-DEGs or TP-DNGs. In addition, if the primary adjacent DNB genes of the core DNB gene were TP-DEGs or TP-DNGs, they would be selected as candidate DNB genes.

### Risk estimation for skin damage by core DNB genes

Independent gene expression data of core DNB genes with/without USBT2627 (Unilever skin lightening active) under UV challenge were adopted for estimation. The independent data include selected gene expression for placebo group and active group at an early time point (8 h post last UV irradiation) and phenotype (L*) at multiple time points (8 h, 24 h, 72 h, and 10 days post UV irradiation). Totally, 38 samples were collected for validation.

Survival analysis was adopted and modified to validate the prediction potency of the identified genes for L*. For the survival curve of phenotypic changes of skin under UV irradiation, the phenotypic value change of the sample greater than a threshold is defined as the occurrence of an event, and the risk here refers to the proportion of samples with such event occurrence at a time point. In the comparisons for *P*-value of survival analysis, the actual number of events occurring in each group over a given time period was compared with the theoretical number of events assuming a same mortality rate in both groups (null hypothesis).

Samples were divided into the high-expression group (group A) and the low-expression group (group B) according to gene expression, with respective sample size of $N_{A}$ and $N_{B}$, and the total number of samples was N. Sample sizes of both groups at each time point were supposed to be equal, i.e.

$$N_{A_{i}}=N_{A},\;N_{B_{i}}=N_{B}\;\;\;\;\;$$

where $N_{A_{i}}$ is the sample size of group A at time${\;T}_{i}$. At $T_{i}$, the observation of event in each group is $O_{A_{i}}$ and $O_{B_{i}},\;$respectively, and the total observation of damage event is

$$O_{i}=O_{A_{i}}+O_{B_{i}}\;\;\;\;\;$$

The theoretical events in each group at time $T_{i}$ are calculated as follows:

$$E_{A_{i}}=O_{i}*\frac{N_{A_{i}}}{N},\;E_{B_{i}}=O_{i}*\frac{N_{B_{i}}}{N}\;\;\;\;\;$$

The total theoretical events in all time points are as follows:

$$E_{A}=\sum_{i}E_{A_{i}},\;E_{B}=\sum_{i}E_{B_{i}}\;\;\;\;\;$$

And the total observations of events in all time points are as follows:

$$O_{A}=\sum_{i}O_{A_{i}},\;O_{B}=\sum_{i}O_{B_{i}}\;\;\;\;\;$$

If the survival rates are the same for both groups, $\frac{{\left(O_{A}-E_{A}\right)}^{2}}{E_{A}}+\frac{{\left(O_{B}-E_{B}\right)}^{2}}{E_{B}}$ would roughly match the chi-square distribution of one degree of freedom, i.e.

$$X^{2}=\frac{{\left(O_{A}-E_{A}\right)}^{2}}{E_{A}}+\frac{{\left(O_{B}-E_{B}\right)}^{2}}{E_{B}}∼\chi^{2}\left(1\right)\;\;\;\;\;$$

where $\chi^{2}\left(1\right)$ is a chi-square distribution with one degree of freedom.

### In vitro validation of tipping point using USBT2627

Since the identified core DNB genes accurately predicted the lightness (L*), a follow-up independent *in vitro* validation using USBT2627 was conducted. Here, USBT2627 was applied after the tipping point as the compared group (Supplementary Figure S8). For the first group, USBT2627 was added on Day 4 and Day 6, respectively, and samples were taken at the first hour of Day 5, Day 6, and Day 7; and for the second group, USBT2627 was added after tipping point, i.e. at the first hour of Day 7 and Day 9, and samples were collected at the first hour of Day 8, Day 9, and Day 10. There were three replicates for each treatment.

### Data sharing statement

Codes used in the paper are publicly available on Github https://github.com/ChengmingZhang-CAS/Landscape-DNB-analysis-for-time-series-data-of-LSE-model-irradiated-by-UVB. Please contact the authors if interested in the data.

## Supplementary material

Supplementary material is available at *Journal of Molecular Cell Biology* online.

## Supplementary Material

mjab060_Supplementary_Material
