# Correction to ‘Landscape dynamic network biomarker analysis reveals the tipping point of transcriptome reprogramming to prevent skin photodamage’

**DOI:** 10.1093/jmcb/mjac013

**Published:** 2022-04-05

**Authors:** Chengming Zhang, Hong Zhang, Jing Ge, Tingyan Mi, Xiao Cui, Fengjuan Tu, Xuelan Gu, Tao Zeng, Luonan Chen

**Affiliations:** State Key Laboratory of Cell Biology, Shanghai Institute of Biochemistry and Cell Biology, Center for Excellence in Molecular Cell Science, Chinese Academy of Sciences, Shanghai 200031, China; University of Chinese Academy of Sciences, Chinese Academy of Sciences, Beijing 100049, China; Unilever Research & Development Centre Shanghai, Shanghai 200335, China; State Key Laboratory of Cell Biology, Shanghai Institute of Biochemistry and Cell Biology, Center for Excellence in Molecular Cell Science, Chinese Academy of Sciences, Shanghai 200031, China; Unilever Research & Development Centre Shanghai, Shanghai 200335, China; Unilever Research & Development Centre Shanghai, Shanghai 200335, China; Unilever Research & Development Centre Shanghai, Shanghai 200335, China; Unilever Research & Development Centre Shanghai, Shanghai 200335, China; Bio-Med Big Data Center, Shanghai Institute of Nutrition and Health, University of Chinese Academy of Sciences, Chinese Academy of Sciences, Shanghai 200031, China; State Key Laboratory of Cell Biology, Shanghai Institute of Biochemistry and Cell Biology, Center for Excellence in Molecular Cell Science, Chinese Academy of Sciences, Shanghai 200031, China; School of Life Science and Technology, ShanghaiTech University, Shanghai 201210, China; Key Laboratory of Systems Health Science of Zhejiang Province, Hangzhou Institute for Advanced Study, University of Chinese Academy of Sciences, Chinese Academy of Sciences, Hangzhou 310024, China; Guangdong Institute of Intelligence Science and Technology, Zhuhai 519031, China

*Journal of Molecular Cell Biology* (2021), 13(11), 822–833, https://doi.org/10.1093/jmcb/mjab060

In the originally published version of this article (p.822), there was an error in the second affiliation of author Chengming Zhang. The erroneous affiliation was given as ‘University of the Chinese Academy of Sciences’. This has now been corrected to ‘University of Chinese Academy of Sciences’.

