# Supplementary material for: Landscape dynamic network biomarker analysis reveals the tipping point of transcriptome reprogramming to prevent skin photodamage
Source: J Mol Cell Biol. 2021 Oct 5;13(11):822–33. doi: 10.1093/jmcb/mjab060 (PMC8782598; doi:10.1093/jmcb/mjab060)
Supplement: mjab060_Supplementary_Material [file mjab060_supplementary_material.pdf]

Supplementary material

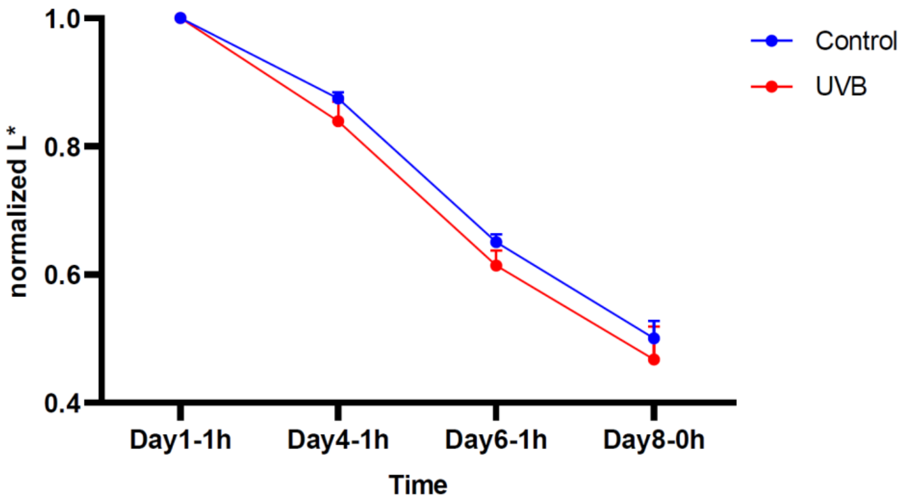

Supplementary Figure S1: The normalized L* value change between Control and UVB groups. L* value was normalized by baseline. Blue: control group; Red: UVB group.

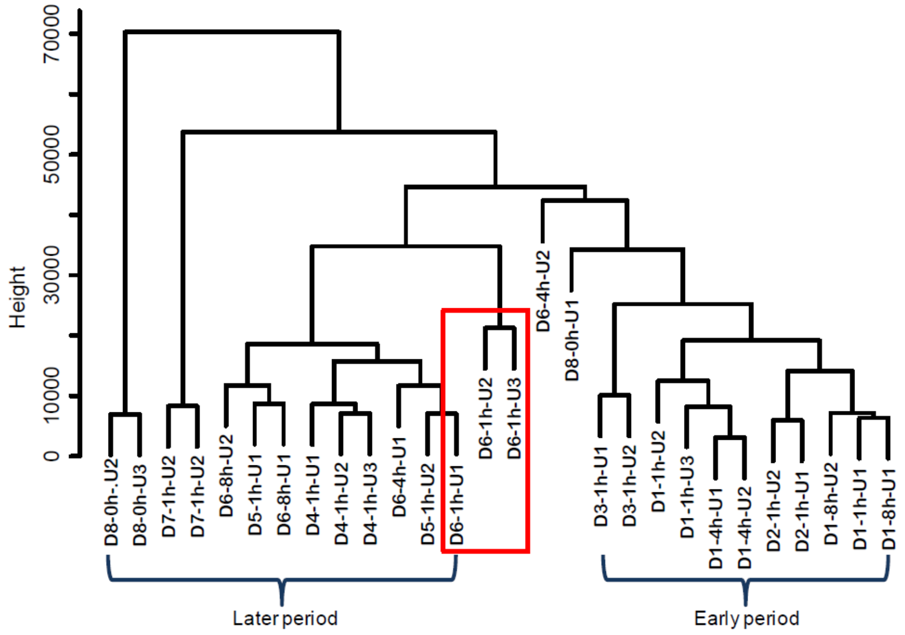

Supplementary Figure S2: Hierarchical clustering analysis for samples in UVB group. D1-1h-U represents the sample of UVB group at 1st hour of Day 1, short for Day 1-1 hour-UVB. The samples at 1 ~ 3 days and 4 ~ 8 days in UVB group can be roughly classified into two categories, namely “Early period” and “Later period”.

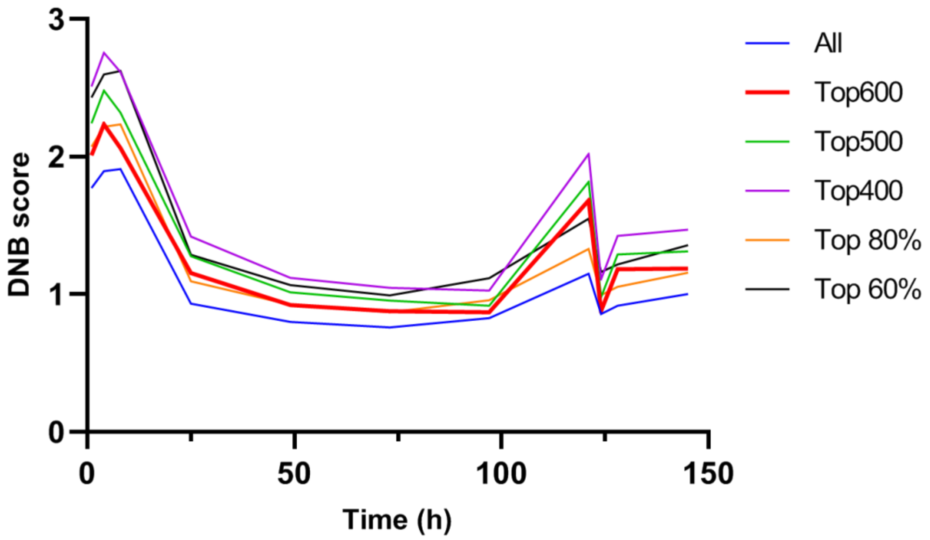

Supplementary Figure S3: DNB scores using genes screened based on different criteria. Blue: DNB score calculated by incorporating all genes in SSN at each time point. Red: DNB score calculated by incorporating top 600 genes in SSN at each time point. Green: DNB score calculated by incorporating top 500 genes in SSN at each time point. Purple: DNB score calculated by incorporating top 400 genes in SSN at each time point. Orange: DNB score calculated by incorporating top 80% of all genes in SSN at each time point. Orange: DNB score calculated by incorporating top 60% of all genes in SSN at each time point. The rank of genes was dependent on individual gene's local DNB score in each SSN.

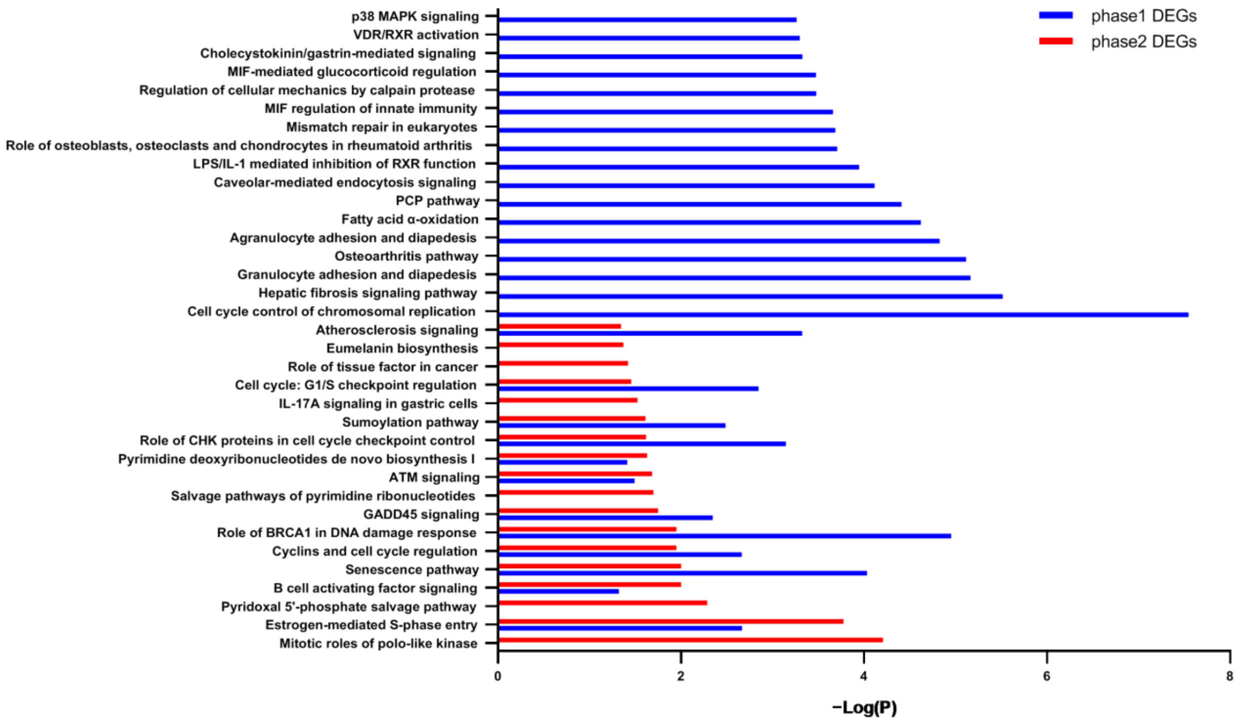

Supplementary Figure S4: Pathways involved by TP-DEGs of phase 1 and phase 2. Phase 1 referred to the first day, which is an acute response to UVB exposure. And phase 2 was the first hour of Day 6, which was an adaptation phase before phenotype change. phase1-DEGs: differential expressed genes of Day1 against Day2-1h and Day3-1h in UVB groups. phase2-DEGs: differential expressed genes of Day6-1h against Day6-4h and Day6-8h in UVB group. −Log(P) is the pathway enrichment analysis involvement significance through IPA.

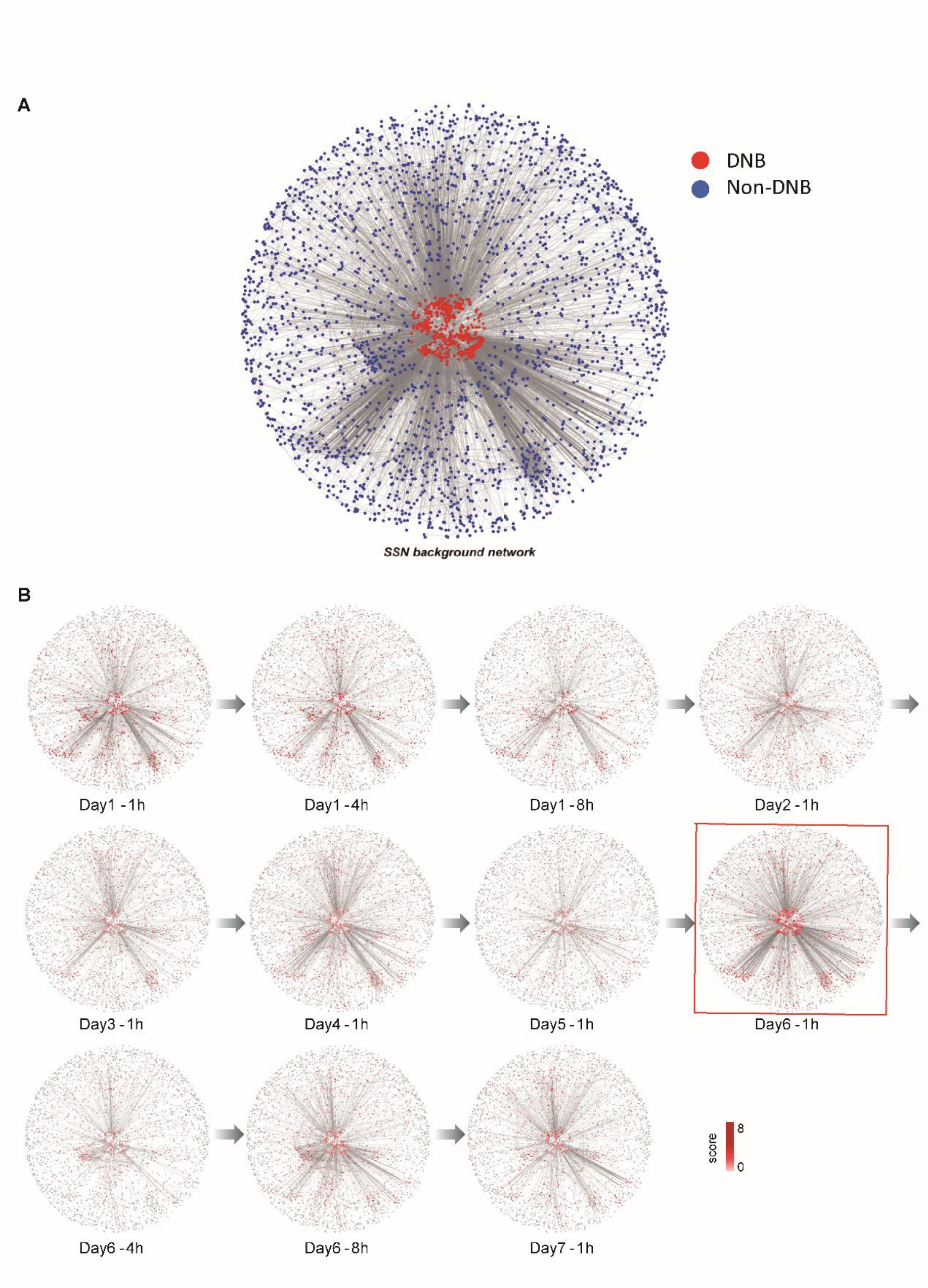

Supplementary Figure S5: The temporal change of single-sample networks induced by UVB irradiation. (A) SSN background network structure. The red points are DNB genes in the background network. (B) SSNs overview at all time points. Deeper red: higher DNB score; Lighter red: smaller DNB score.

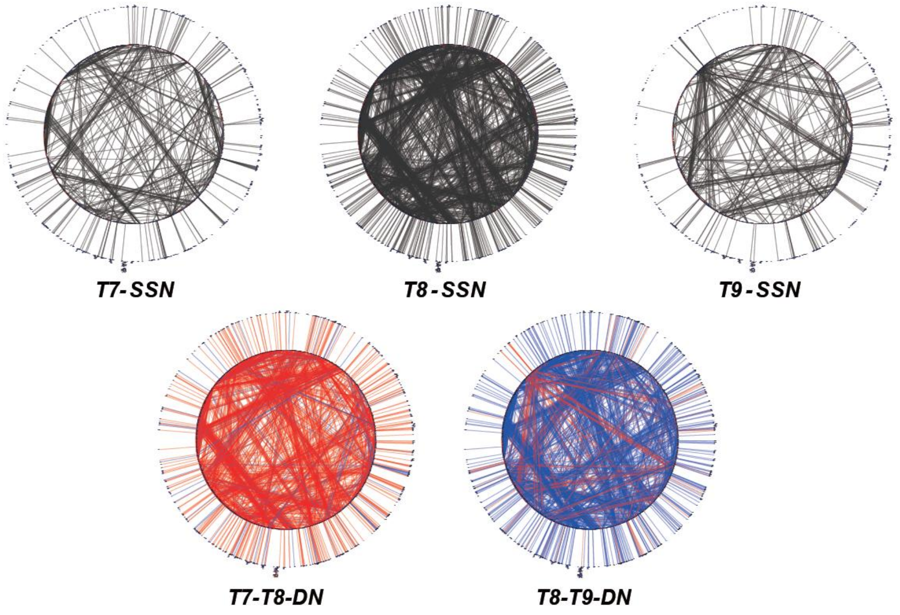

Supplementary Figure S6: Differential networks at time point around tipping point. The above three graphs are single sample networks at the pre-tipping point (T7: Day 5-1h), tipping point (T8: Day 6-1h) and post-tipping point (T9: Day 6-4h). T7-T8-DN: differential network between pre-tipping point (T7: Day 5-1h) and tipping point (T8: Day 6-1h); T8-T9-DN: differential network between tipping point (T8: Day 6-1h) and post-tipping point (T9: Day 6-4h). Red: new appeared edges; Blue: disappeared edges.

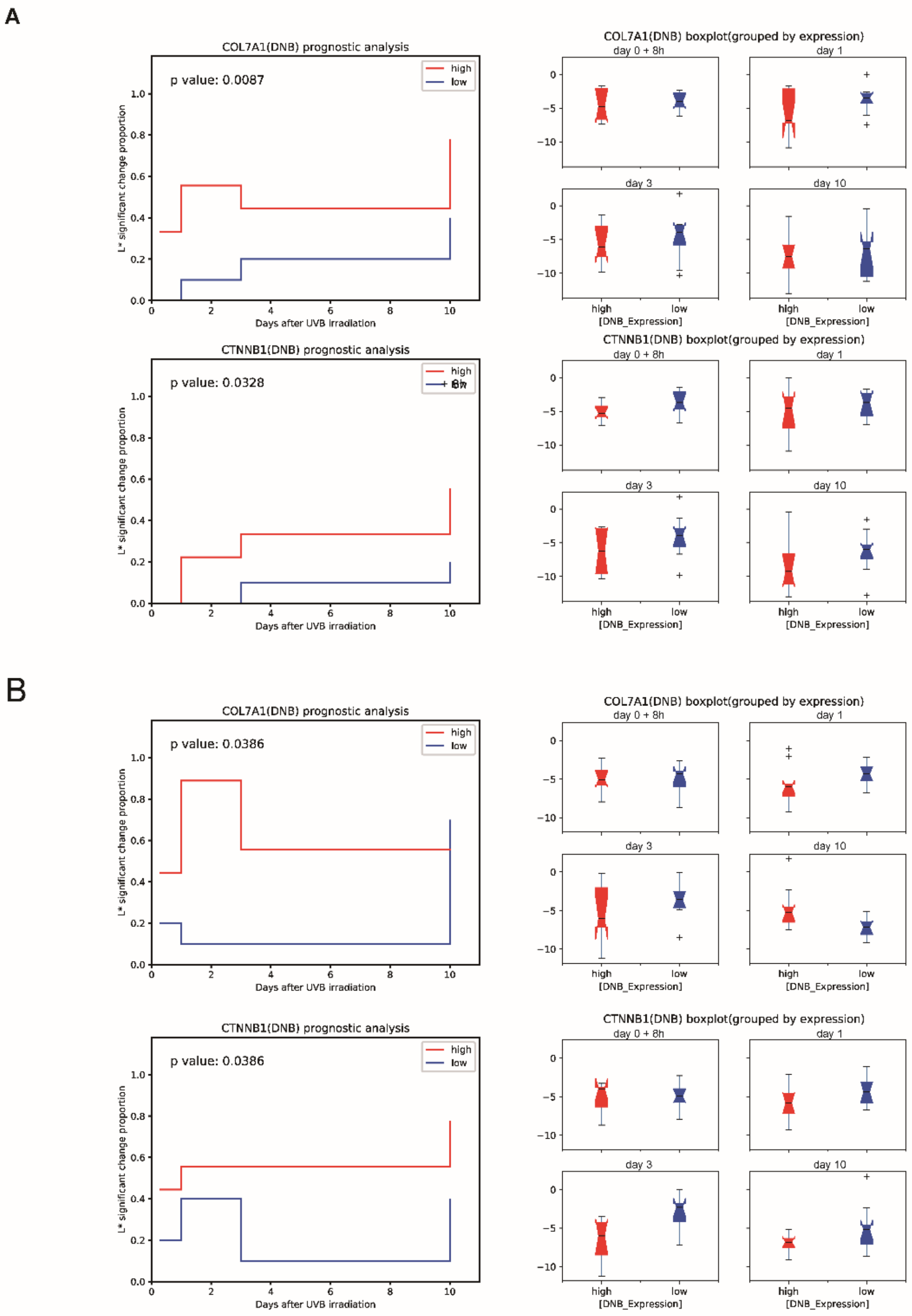

Supplementary Figure S7: **The assessment of core DNB genes on skin data.** (A) Core DNB prognostic analysis on clinical placebo group (L*****); (B) Core DNB prognostic analysis on clinical active group (L*****).

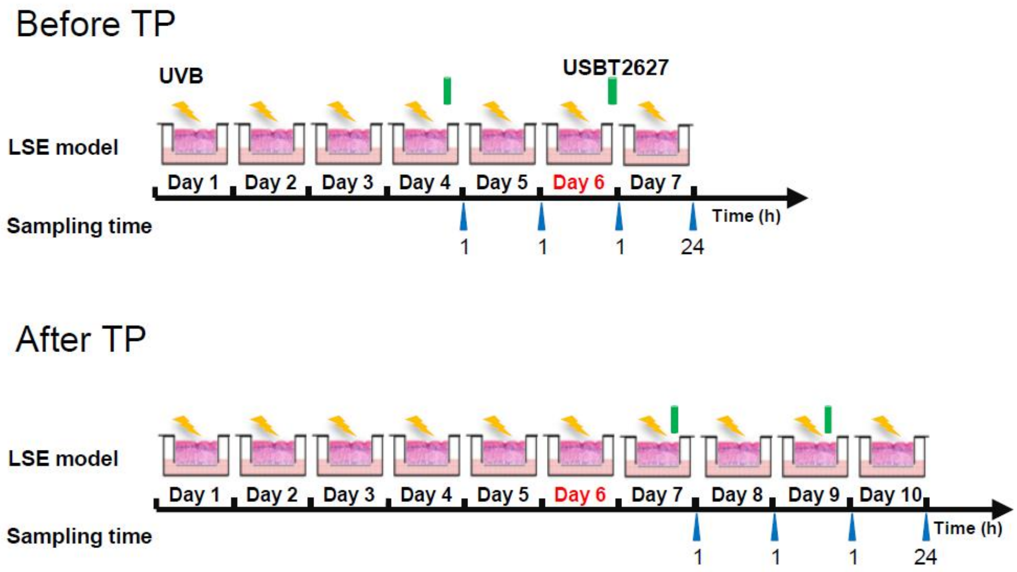

Supplementary Figure S8: Experiment design of independent in vitro study to demonstrate the importance of skin protection efficacy of treatment before tipping point. Before TP: USBT2627 was applied at Day 4. After TP: USBT2627 was applied at Day 7.

Supplementary Table S1. The prognostic significance of core DNB gene using different thresholds in placebo group

	**Threshold=0.4**	**Threshold=0.5**	**Threshold=0.6**	**Threshold=0.7**
PCNA	0.308224	0.192792	0.079411	0.052537
ITGB1	0.047121	0.06579	0.032828	0.022119
SFN	0.515677	0.50828	0.306197	0.279879
CALML5	0.308224	0.36304	0.52011	0.464786
COL7A1	0.047121	0.008655	0.306197	0.633553
HNRNPD	0.308224	0.036805	0.230666	0.181926
PSMB10	0.308224	0.605288	0.930111	0.898839
COL4A2	0.985983	0.605288	0.930111	0.898839
CTNNB1	0.098655	0.06579	0.032828	0.022119
TGFB1	0.75183	0.788128	0.52011	0.464786
ERCC2	0.985983	0.901288	0.079411	0.052537
BRCA1	0.985983	0.901288	0.306197	0.633553
NFKB1	0.176135	0.036805	0.079411	0.052537

Supplementary Table S2. The prognostic significance of core DNB gene using different thresholds in active group

	Threshold=0.4	Threshold=0.5	Threshold=0.6	Threshold=0.7
PCNA	0.938721	0.681846	0.267948	0.052537
ITGB1	0.61188	0.95542	0.267948	0.181926
SFN	0.829567	0.173743	0.114289	0.279879
CALML5	0.829567	0.765594	0.832004	0.279879
COL7A1	0.166421	0.038606	0.114289	0.279879
HNRNPD	0.274982	0.514281	0.257899	0.279879
PSMB10	0.938721	0.95542	0.832004	0.464786
COL4A2	0.423985	0.765594	0.257899	0.464786
CTNNB1	0.166421	0.038606	0.114289	0.022119
TGFB1	0.508518	0.444873	0.267948	0.052537
ERCC2	0.274982	0.08647	0.494268	0.279879
BRCA1	0.61188	0.765594	0.813664	0.898839
NFKB1	0.938721	0.514281	0.257899	0.464786

Supplementary Table S3. Gene information

Gene symbol	Gene full name
BRCA1	breast cancer type 1 susceptibility protein
CALML5	calmodulin like 5
COL1A1	collagen type I alpha 1 chain
COL4A1	collagen type IV alpha 1 chain
COL7A1	collagen type VII alpha 1 chain
COMP	cartilage oligomeric matrix protein
CTNNB1	catenin beta 1
DCT	dopachrome tautomerase
E2F4	E2F transcription factor 4
ERCC2	ERCC excision repair 2, TFIIH core complex helicase subuni
HMGCS1	3-hydroxy-3-methylglutaryl-CoA synthase 1
HNRNPD	heterogeneous nuclear ribonucleoprotein D
ITGB1	integrin subunit beta 1
MMP	matrix metalloproteinase
MYC	MYC proto-oncogene, bHLH transcription factor
NF-κB	nuclear factor kappa B subunit
PCNA	proliferating cell nuclear antigen
PSMB10	proteasome 20S subunit beta 10
SFN	stratifin
TGFB1	transforming growth factor beta 1
TIMP1	TIMP metallopeptidase inhibitor 1
TP53	tumor protein p53
TWIST1	twist family bHLH transcription factor 1
VEGFA	vascular endothelial growth factor A
